# Rheological comparison of sputum and reconstituted airway epithelium mucus

**DOI:** 10.1038/s41598-024-80932-y

**Published:** 2024-12-30

**Authors:** Lydia Esteban Enjuto, Vassylia Taty Poaty, Mendy Bouveret, Huang Song, Samuel Constant, Jérémy Patarin

**Affiliations:** 1 Rheonova, 1 Allee de Certéze, 38610 Gières, France; 2 Epithelix, CH-1228 Plan-les-Ouates, Geneva, Switzerland

**Keywords:** Biophysics, Structural biology, Medical research, Materials science, Physics

## Abstract

Pulmonary mucus serves as a crucial protective barrier in the respiratory tract, defending against pathogens and contributing to effective clearance mechanisms. In Muco Obstructive Pulmonary Diseases (MOPD), abnormal rheological properties lead to highly viscous mucus, fostering chronic infections and exacerbations. While prior research has linked mucus viscoelasticity to its mucin content, the variability in MOPD patients implies the involvement of other factors. To isolate these effects, mucus produced by epithelia reconstituted in vitro serves as a powerful versatile model for mucin research. This study characterises the rheology of mucus collected from Air-Liquid Interface (ALI) cultures and compares it to sputum samples from MOPD patients, demonstrating that macrorheology with cone-plate geometries is a reproducible method for analysing small mucus quantities from ALI cultures. While sputum samples exhibit similarities in rigidity with ALI mucus, they also display structural differences and variations in their response to substantial deformations. The study highlights the importance of understanding mucus behaviour under large deformations, emphasising the role of ALI cultures as a controlled environment for conducting detailed studies.

## Introduction

Pulmonary mucus serves as a protective barrier, and an innate defence mechanism against bacteria, dust and viruses. At the same time, it contributes to the hydration of the respiratory tract, facilitating effective clearance mechanisms^1^. In Muco Obstructive Pulmonary Diseases (MOPD), such as Cystic Fibrosis (CF), Chronic Obstructive Pulmonary Disease (COPD), Non-Cystic Fibrosis Bronchiectasis (NCFB) or chronic asthma, mucus is highly viscous due to abnormal rheological properties and subsequent mucus accumulation on the small airways^2–4^. In such conditions, pathogens can proliferate in the trapped mucus, generating chronic infections and exacerbations^5^, and eventually leading to hospitalisation and increased mortality^6,7^.

Previous research has identified a direct correlation between mucus viscoelasticity and its mucin content^2,3,8^. Nevertheless, this correlation exhibits variability in MOPD patients, potentially attributed to other elements within the mucus. Notably, individuals experiencing exacerbations show increased viscoelasticity in their sputa^9^ which has been associated to the presence of bacteria^10,11^, and inflammatory markers^12^. Nonetheless, it is surprising that the majority of the publications on mucus and sputum rheology tend to focus solely on its viscoelasticity at rest, rather than examining its response under strong efforts that more closely emulate mucus transport conditions and pathogen penetration^1^. Indeed, recent data by Volpato et al.^13^ revealed a positive correlation between critical shear stress and sputum eosinophilia in MOPD patients which has also been related to low FEV_1_ and mucus plugging^14^. Additionally, large stresses play a pivotal role in mucus removal, as the different clearance strategies need to overcome mucus adhesion and cohesion^15^ disrupting the mucus gel. Current approaches focus on either fluidifying the mucus or applying vibrations to enhance mucus transport. Pharmacological treatments that disrupt the mucus cross-linked network, such as N-acetylcysteine, which breaks disulphide bonds, or recombinant human DNase^16^, have shown a significant reduction in the critical shear stress of CF sputum after treatment^17,18^. In contrast, airway clearing devices, chest physiotherapy, and exercise promote mucus removal by subjecting it to medium and large strains and stresses^19^, facilitating easier expulsion through cough, which has been associated to shear stress values up to 100 Pa in the bronchi^1,15^.

Understanding mucus at a molecular and rheological level can aid in developing targeted therapies and designing more effective drug delivery systems. While sputum and mucus collected in vivo reflect the real-time condition in living individuals, model mucus provide a controlled environment for more detailed studies under laboratory conditions. Diverse mucus models cater to various scientific objectives in research. Artificial mucus models from synthetic materials create controlled settings for the precise exploration of rheological properties, allowing researchers to tune physical conditions. Cell culture models enable the investigation of cell-mucus interactions and mucociliary clearance in vitro, offering insights into cellular responses. Certain animals, such as fish or snails^1,20^, present an opportunity to examine mucus within the broader context of a complete biological system. However, each model has its limitations, including artificial mucus lacking the full complexity of native mucus, cell culture models oversimplifying in vivo conditions, and animal models exhibiting compositional and structural differences.

Throughout this article, we have focused on characterising the mucus produced ex vivo through Air-Liquid Interface. (ALI) cell culture. ALI cultures yield sterile mucus resembling “healthy” samples, allowing for precise adjustments to its viscoelastic properties by modifying solid concentration. This controlled “healthy” environment can subsequently be modified by introducing pathogens, particles, or inflammatory mediators, facilitating the comprehensive study of mucus evolution.

In rheometry, the use of plate-plate measurement cells is widespread for assessing heterogeneous samples like sputum. However, these geometries require a substantial mucus volume of at least 330 µL. Given the limited mucus obtained from ALI cultures, alternative methods are necessary. Although particle tracking microrheology (PTMR) has been utilised in mucus measurements^8,21^, it often entails specialised microscopic equipment and tracking particles, adding complexity, costs, and concerns about interactions between the sample and seeded particles. Furthermore, microrheology only provides insights into the local mechanical properties of mucus, whereas macrorheology reveals the overall rheological characteristics of the sample. Another option is employing cone-plate geometries, which considerably reduces the required sample volume for macro-rheological measurements. The only limitation of these geometries is their unsuitability for heterogeneous samples, as the sample may not uniformly respond to shear, and continuous mechanics principles may not apply^22^. ALI mucus, being homogeneous, makes cone-plate geometries perfectly suitable.

In this paper, we detail the procedure for collecting and testing the rheology of mucus from the epithelium surface. We then explore the rheological behaviour of ALI mucus compared to sputum samples from MOPD patients to assess the value of using ALI mucus in the development of treatments for obstructive diseases.

## Materials and methods

### ALI cultures

ALI cultures used in this study are Epithelix’ proprietary technology MucilAir™. MucilAir™ is a pseudostratified and ready-to-use 3D model of human airway epithelium, constituted with primary human epithelial cells freshly isolated from nasal, tracheal, or bronchial biopsies. MucilAir™ respiratory epithelium contains approximately 500,000 cells for a surface area of 0.33 cm^2^. When switched at the ALI, the progenitor cells undergo progressive differentiation and polarisation into a fully ciliated epithelium over a period of 35 days. The mature MucilAir™ is composed of basal cells, ciliated cells and mucus cells. The proportion of these various cell types is preserved compared to what one observes in vivo^23^. Moreover, MucilAir™ is functionally differentiated and secretes mucus.

Mucus from MucilAir™ was collected by centrifugation allowing the integrity of the epithelium to be maintained. Each insert was inspected under a conventional inverted microscope to ensure the quality of the epithelia and to detect the presence of mucus by visually examining the refractive aspect of the apical surface. MucilAir™ were transferred into sterile 50 ml centrifuge tube, membrane to the top followed by a centrifugation at 2000 rpm or 650 g during 5 min. The collected mucus was immediately transferred into a microcentrifuge tube and pooled with other centrifugation process on several cultures until sufficient volume was reached (> 30 µl). The total volume produced per culture was estimated at approximately 4 µl.

We have analysed 43 mucus samples produced in vitro by healthy nasal epithelia (n = 32) and bronchial epithelia from healthy (n = 11) cells and 7 mucus samples produced by cells from CF donors.

### Sputum samples

#### CF and NCFB sputum collected by autogenic drainage

Sputum samples were collected at the ‘Centre de Ressource et de Compétence de Mucoviscidose’ in the Grenoble Alpes University Hospital from CF and NCFB patients in the context of an observational monocentric study declared to the national authority (ANSM) under RCB 2020-A02367-32. Research was performed in accordance with the relevant guidelines and regulations. Written informed consent was obtained from all participating patients, and their clinical details were anonymized and treated confidentially. The study received approval from the French research ethics committee (Comité de Protection des Personnes, case number 20.09.08.61213). Detailed protocol and eligibility criteria are provided in clinicaltrials.gov under NCT04687319.

Under the guidance of a physiotherapist, sputum samples were induced by autogenic drainage^19^ and collected in sterile flat-bottom tubes. After expectoration, the saliva is located above the bronchial secretions due to the presence of bubbles. Within an hour of collection, a positive displacement pipette (PosD MR-1000, Mettler Toledo) was carefully submerged below the saliva layer, near the bottom of the sample. The bronchial secretions were aspirated, while the foam (saliva) remained in the original tube. Mucus was transferred to sterile tubes and homogenised by gradually increasing vortex stirring until it visually formed a torus. This vortex intensity was maintained for 30 s, resulting in visually homogeneous samples. In this study 81 expectorations were collected from 59 CF and 22 NCFB adult patients. Collected volumes varied across patients and diseases from 0.4 ml to 7 ml. When sufficient, samples were divided into 500 µl aliquots, then freeze dried and stored at -80°C. Before rheological measurements, samples were thawed at 37°C. This sample handling protocol was previously validated and published^24^.

#### Induced healthy sputum

To provide a comparison with the mucus samples produced by healthy cells in ALI cultures, we included rheological data from 11 induced healthy sputum samples from adult volunteers published by Patarin et al.^4^. Since healthy individuals do not naturally produce sufficient amounts of sputum, induction is necessary to collect these samples.

#### Induced CF sputum

To investigate the effect of induction on sputum rheology, we additionally considered the rheological data of induced 18 CF sputum samples reported by Patarin et al.^4^.

### Rheometry

Rheological assessment was conducted using the Rheomuco rotating rheometer from Rheonova, France. The rheometer was equipped with rough plate-plate geometries of 25 mm diameter for sputum and cone-plate geometries for ALI mucus. The choice of two geometries reflects the need to accommodate the different sample types: ALI mucus is always in small volumes, while sputum samples require larger gaps due to their heterogeneities. Importantly, no differences in the results occur due to the geometries used, as the rheometer automatically compensates for the specific dimensions of each geometry in its calculations. Measured volumes were 86 µl for ALI mucus and between 330 and 690 µl for sputum.

The samples were subjected to increasing oscillatory strain amplitudes ranging from 0.1 to 10,000% at 1 Hz and 37 °C. Throughout this article, we explore two variables in the viscoelastic linear domain (low strains) that characterise mucus behaviour at rest, and two in the non-linear domain (high strains) to elucidate its behaviour during flow (see Table 1). The viscoelastic modulus G* is the complex resultant of the elastic and viscous moduli G′ and G″, indicating the overall consistency of the sample at rest. The damping factor tan δ provides insights into the molecular network morphology; a lower value implies a more solid-like microstructure, where the mucus exhibits greater internal friction and dissipates energy more efficiently. In the large deformation domain, we determined the critical point at which flow begins: the critical strain γ_c_ is calculated at the intersection of G′ and G″, which occurs at the critical modulus G_c_. We then calculated the corresponding critical stress σ_*c*_, indicating the stress required for the onset of flow.

**Table 1:** Definitions and units of rheological parameters.

Domain	Parameters	Unit	Signification
Small deformation	Viscoelastic modulus ${\bf G^*} = \sqrt {G^{\prime } {^2} + G^{{\prime \prime }} {^2}}$	Pa	Overall resistance to deformation. The higher the G*, the more consistent
	Damping factor **tan δ** = G″/G′	–	Factor related to the nature of the structure. Tan δ = 0: solid; tan δ < 1: gel; tan δ > 1: liquid
Large deformation	Critical strain **γ**_**c**_	–	Gel extensibility: deformation required to start flow. Low: brittle; high: extensible
	Critical stress **σ**_**c**_ = 2G_c_γ_c_	Pa	Gel strength: force required to initiate flow. Low: fragile; high: ductile

### Statistics

We performed statistical analysis using Matlab R2023b. Normality was assessed through the Shapiro-Wilk test, and group comparisons were made using unpaired two-tailed t-tests.

## Results

### Sputum rheology

**Fig. 1:**
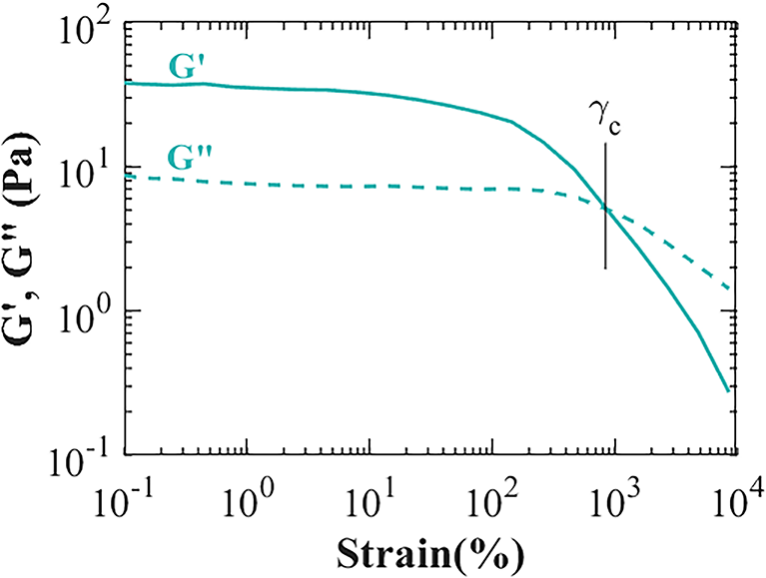
Strain sweep of a CF sputum sample. The solid line is the elastic modulus (G′) and the dashed line is the viscous modulus (G″). The critical strain (γ_c_) is retrieved when G′ and G″ cross.

All the samples measured exhibited similar behaviours to the curve in Fig. 1. Under low strain conditions, both the elastic (G′) and viscous modulus (G″) remained constant, with G′ exceeding G″, indicating a predominantly elastic nature in the sputum. In this region, sputum responded linearly to the applied deformation. As the strain continues to increase, the response becomes nonlinear, leading to an evolution of G′ and G″ until reaching the critical deformation γ_c_ and critical stress σ_c_. At this critical point, G″ surpasses G′, marking the transition from elastic to viscous behaviour, indicating that the sample can begin to flow beyond this point.

The rheological parameters exhibited log-normal distributions, consistent with previous publications^4,24^. Table 2 displays the geometric mean values for CF and NCFB sputum rheology, along with their confidence intervals CI at an alpha level of 0.05. The CF and NCFB low strain data shows G* values of 9.4 and 8.6 Pa respectively and a consistent tan δ of 0.27 for both groups, indicating a predominantly elastic gel behaviour. At the flow point, σ_c_ was 27.3 Pa for CF and 31.3 Pa for NCFB, while γ_c_ was 12.5 for CF and 15 for NCFB, showing low variability within the populations. No significant differences were found between the rheological properties of these diseases (*p*-value > 0.1), supporting earlier observations^24^. Consequently, we combined all the sputum samples into a single population for subsequent analysis.

**Table 2:** The table includes rheological data for CF and NCFB sputum and ALI mucus collected from bronchial and nasal cell cultures, with respective sample sizes (n).

			Low strain		Flow point	
	Sample	n	G* (Pa)	tan δ [–]	σ_c_ (Pa)	γ_c_ [–]
**Sputum**	CF	59	9.4 [7.2, 12.1]	0.27 [0.26, 0.29]	27.3 [22.3, 33.3]	12.5 [11.1, 14.2]
	NCFB	22	8.6 [6.2, 12.1]	0.27 [0.25, 0.29]	31.3 [23.8, 41.2]	15.0 [12.3, 18.4]
	*p*-value		0.6971	0.5856	0.4165	0.1245
**Cell cultures**	Bronchial	11	5.9 [1.7, 20.3]	0.39 [0.25, 0.59]	3.9 [0.9, 16,9]	0.7 [0.2, 2.6]
	Nasal	32	6.1 [3.2, 11.3]	0.48 [0.38, 0.61]	1.3 [0.7, 2.6]	0.5 [0.3, 0.8]
	*p*-value		0.9603	0.3681	0.1596	0.5579

The values are presented as geometric means and 95% confidence interval CI [lower CI, upper CI]. The p-values are provided for statistical comparisons for each rheological parameter.

#### Influence of sputum collection method: induction versus autogenic drainage

**Fig. 2:**
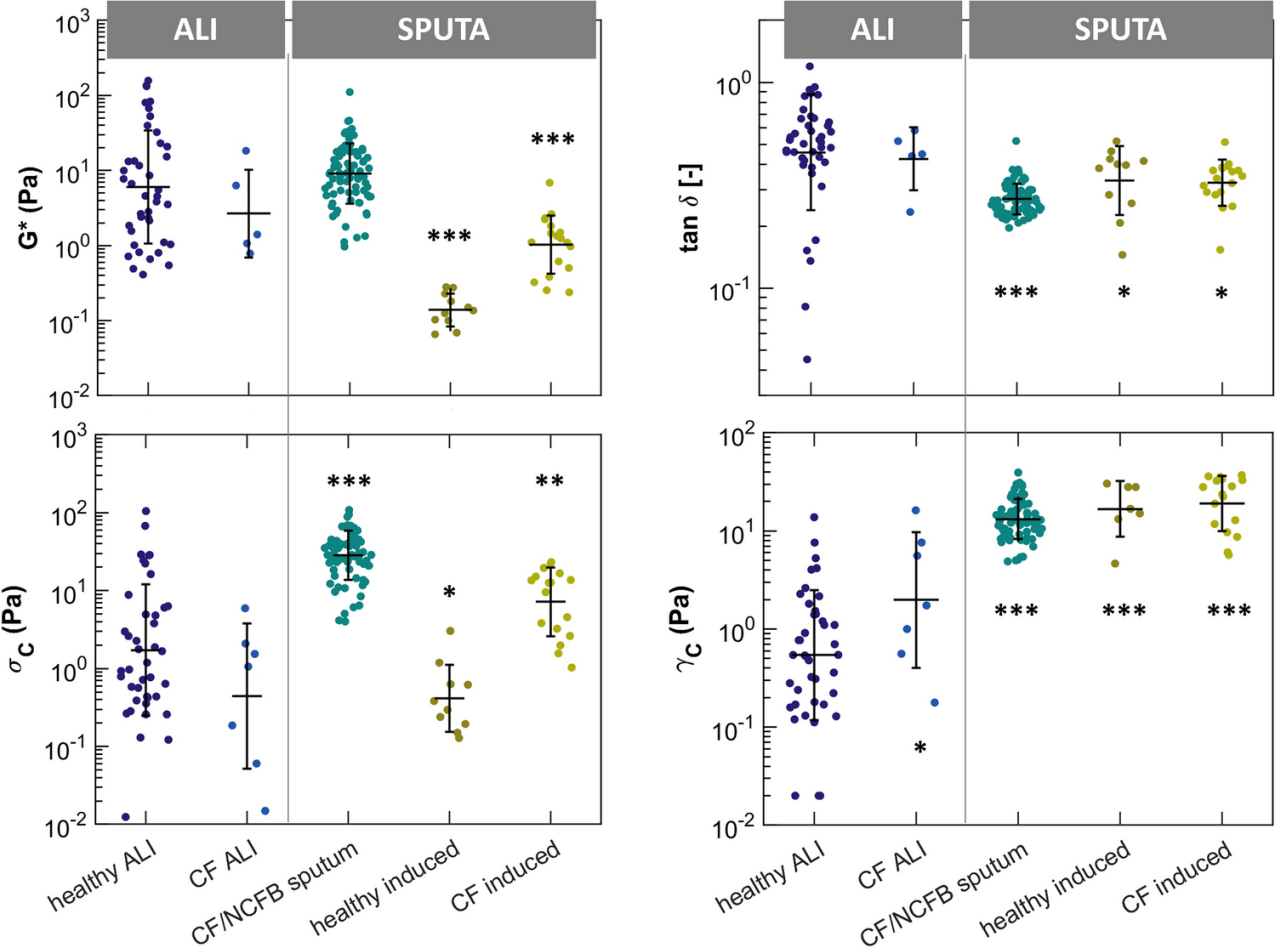
Rheological parameters G*, tan δ, σ_c_ and γ_c_ measured from ALI mucus samples from healthy donors (N = 43) and CF donors (N = 7); as well as sputum collected by auto drainage from CF and NCFB patients (N = 81) and induced sputum from healthy individuals (N = 11) and CF patients (N = 18). Statistical significance is marked as follows: ***p < 0.001, **p < 0.01, *p < 0.05 compared to healthy ALI mucus.

The rheological comparison between CF sputum and CF-induced sputum revealed a pronounced dilution effect in the induced samples which is observable in reductions of G* and σ_c_ (Fig. 2). Sputum is hydrated and its mesh swells. Specifically, G* was reduced by an order of magnitude, decreasing from 9 Pa in CF sputum to 1 Pa in CF-induced sputum (*p* < 0.0001). This reduction is consistent with previous research that has established G* to be concentration-dependent^2,27^, reinforcing our observation of dilution effects in the induced samples. Similarly, the critical stress σ_c_ dropped by a factor of 4 from 28 Pa to 7 Pa (*p* < 0.0001), highlighting the diminished resistance to large deformations in the induced samples.

### ALI mucus rheology

The logarithmic values of G*, tan δ, σ_c_ and γ_c_ measured in ALI mucus also followed normal distributions (*p* > 0.05). The rheological characteristics of ALI mucus derived from bronchial and nasal cell cultures were strikingly similar. As displayed in Table 2, the p-values indicate that there were no statistically significant differences between bronchial and nasal cultures for any of the rheological parameters (*p* > 0.1), indicating that the mechanical properties of mucus were similar regardless of its origin. This similarity implies that, despite the anatomical differences between the bronchial and nasal cells, the mucus produced in vitro behaves similarly under stress and strain conditions.

Altogether, healthy ALI mucus exhibited mean values of G* = 6 Pa, tan δ of 0.46, σ_c_ of 1.7 Pa and γ_c_ of 65%. It is noteworthy to highlight that within the ALI distributions, certain data points deviated significantly from the rest of the measurements leading to a large dispersion (Fig. 2). This increased variability may reflect the inherent heterogeneity of in vitro systems, where cell cultures can differ in maturity, differentiation states, or environmental conditions.

### ALI mucus exhibits lower extensibility and increased solid-like structure

The comparison between sputum and ALI mucus reveals some notable differences in rheological properties, as illustrated in Fig. 2. We found large disparities in γ_c_ with all sputum samples displaying much higher critical strain values averaging over 1000%, compared to just 65% in healthy ALI mucus (*p* < 0.0001). This higher extensibility indicates that sputum can withstand greater deformation before breaking. Furthermore, tan δ was significantly lower for all sputum samples, reflecting a more pronounced solid-like behaviour compared to ALI mucus. Therefore, sputum has a greater capacity to store energy, likely due to a higher degree of crosslinking, making it more elastic when subjected to external forces. In contrast, excluding the dilution effect, no significant difference was found in G* between ALI mucus and CF and NCFB autogenic drained sputum, with mean values of 6 and 9 Pa respectively (*p*-value = 0.137), suggesting that the overall viscoelasticity of both is comparable.

**Fig. 3:**
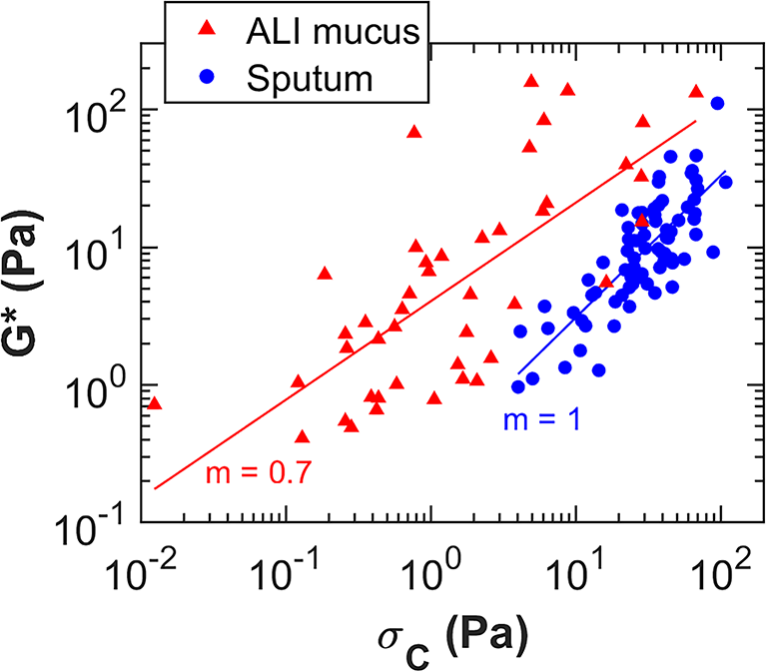
Correlation between σ_c_ and G* for the 81 CF and NCFB sputum samples (blue circles) and the 50 healthy and CF ALI mucus samples (red triangles), m indicates the slopes of the fits.

Interestingly, we observed a consistent positive relationship between the functional variables G* and σ_c_ with similar slopes in both the mucus and sputum populations, as depicted in Fig. 3. The higher the viscoelasticity of a sample, the greater the required stress to make the mucus flow. Moreover, the comparable slopes in ALI mucus and sputum suggests a common mechanical behaviour governing the interaction between G* and σ_*c*_, despite differences in the origin or nature of these samples. Still, sputum samples present a more pronounced association between the two parameters, with a slope of 1 compared to 0.7 for ALI mucus. As previously mentioned, the viscoelastic modulus G* has already been directly linked to the solid content^2,27^. Figure 3 shows that, at a constant G*, ALI mucus has a lower σ_c_​ compared to sputum. This means that, although both samples have the same solid content, the ALI mucus requires less stress to start flowing, reflecting a simpler internal structure with fewer internal connections compared to sputum.

Regarding the effect of disease, the critical stress σ_c_ was substantially higher in sputum samples from diseased patients, even when comparing those collected by induction (Fig. 2). This further emphasizes the more resilient structure to large deformation of pathological sputum, which is a key characteristic of the airway obstruction commonly observed in MOPDs. In contrast, CF ALI mucus displayed rheological properties similar to those of healthy ALI mucus (Fig. 2), with the exception of a slight but statistically significant increase in extensibility γ_c_ (*p* = 0.045). However, these findings are considered preliminary due to the limited sample size (n = 7), of which two samples did not exhibit a clear plateau in the linear viscoelastic region. Further data are required to draw definitive conclusions about CF ALI mucus rheology.

## Discussion

In our investigation, we do not see significant differences in G* between ALI and sputum samples therefore we can conclude that the amount of solid matter (mainly mucins) is very similar. However, tan δ is lower for sputum samples indicating that sputum has a more solid-like structure. In other words, the network has more connections as tan δ = energy loss/energy storage, therefore sputum is losing less energy and storing it more when a deformation is applied to it. Moreover, the parameters at the critical point indicate at which point we are able to break the non-covalent connexions of the network. The critical stress σ_c_ and critical strain γ_c_ for ALI mucus is significantly lower (over 1 order of magnitude for the mean values). Thus, less deformation and stress are required to break the entangled network and make the sample flow.

The structural differences observed in the rheological assessment may be attributed to variations in the ratios of MUC5B and MUC5AC mucins. Several publications^28,29^ have shown that MUC5B assembles into linear bundles, while MUC5AC forms an intricate, highly branched network. Given that MUC5AC is upregulated in response to infections, sputum from CF and NCFB patients is likely to contain higher levels of MUC5AC compared to mucus from ALI cultures. Consequently, we would also expect differences between healthy sputum and diseased sputum, as the latter would have a greater proportion of MUC5AC, leading to a more complex mucin network structure. However, our results show that both γ_c_ and tan δ remain constant across sputum samples, revealing no significant differences between the healthy and diseased groups. Therefore, the differences observed between ALI mucus and sputum samples do not seem to stem solely from mucin types but should be influenced by the presence of macromolecules such as globular proteins, DNA, actin, bacterial biofilms and inflammatory proteins from neutrophils or eosinophils.

**Fig. 4:**
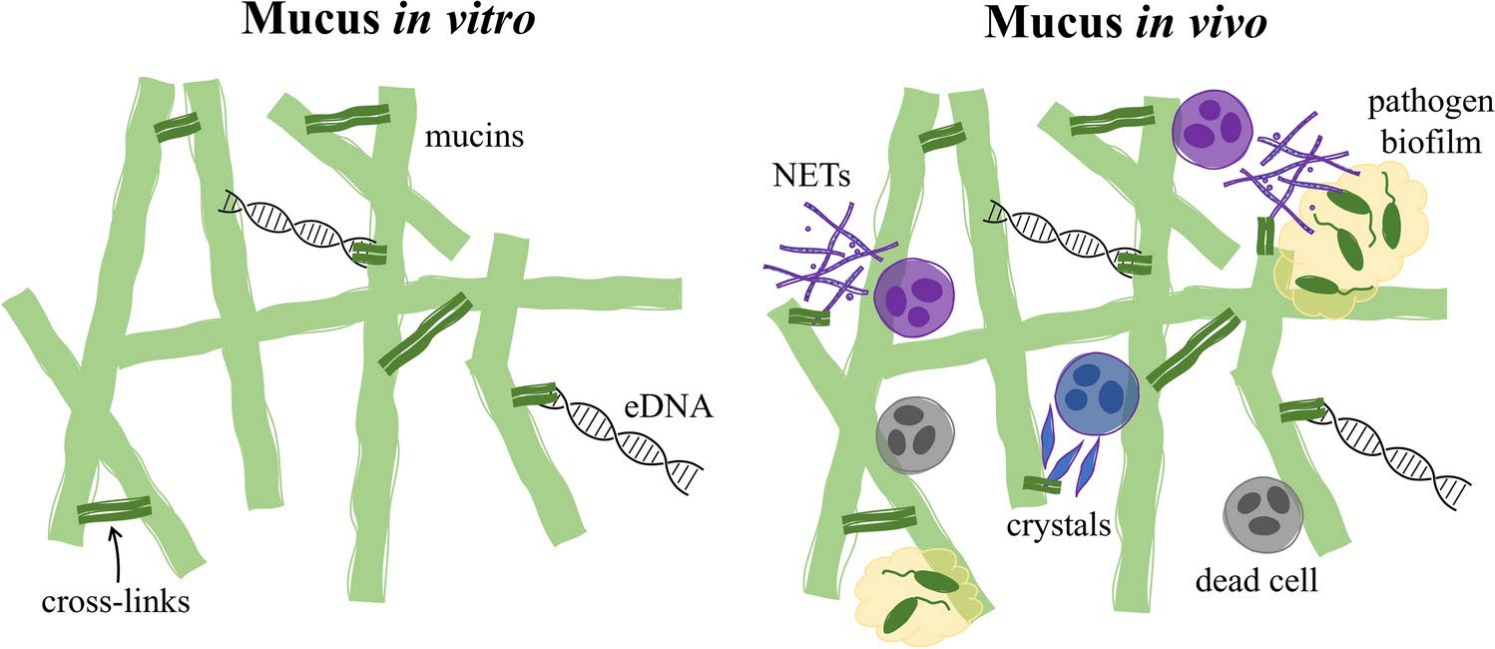
Illustration depicting the cross-linked structure of in vitro mucus, primarily composed of mucins and extracellular DNA. Additionally, in vivo mucus typically incorporates bacteria that can form biofilms, along with immune response cells capable of releasing polymeric materials such as actine polymerised neutrophil extracellular traps (NETs)^18^ and Charcot-Leyden crystals^26^.

We thus propose the structural and compositional differences between mucus in vitro and in vivo depicted in the illustration in Fig. 4. The distinct behaviour of ALI mucus can be attributed to its composition, primarily consisting of mucins and DNA. In contrast, sputum samples, reflecting real-world conditions, encompass a broader range of components, including pathogens, inflammatory mediators, and cell debris. The presence of these additional elements in sputum, as already suggested by Ma et al. and Liu et al.^9,25^, may drastically contribute to a more complex gel structure, characterised by increased bonding and consequently greater gel strength, resulting in a more solid conformation. For instance, Rouillard et al. showed that inoculating Pseudomonas aeruginosa (PAO1) into ALI mucus significantly increased mucus viscoelasticity through biofilm formation^11^. Furthermore, confocal microscopy images of CF sputum^18^ showed the cross-linking of mucins by DNA and neutrophil-released actin bundles. Rheology of the imaged mucus illustrated how it became more fragile when disaggregating them from the structure using heparin, aligning with the effects observed when treating mucus with rhDNAse^17^. In addition, Aegerter et al.^26^ have recently proposed the alterations in mucus rheology could be a result of an increased mucin crosslinking due to oxidations and formation of Charcot-Leyden Crystals (CLCs) released by eosinophils. Moreover, recent work by Batson et al.^5^ found no significant increase in mucin production during exacerbations in CF sputum, despite evident changes in rheology^9^. Therefore, it seems that even though mucins are the primary gel-forming units of mucus, understanding the effects of inflammatory markers and pathogens on the mucus network is crucial for a comprehensive understanding of mucus behaviour. This knowledge is essential for accurate patient follow-up and the selection of targeted treatments.

Although the techniques and concepts presented here are not entirely novel, the comparison of large strain rheology between sputum and ALI samples represents a unique contribution to the field. To our knowledge, no previous studies have specifically explored this aspect. Earlier studies, such as that by Hill et al.^27^, only examined Small Amplitude Oscillatory Shear (SAOS) rheology, which provides insights into the sample’s properties at rest. However, SAOS does not capture the behaviour of mucus under large deformations, which is crucial for understanding its functional response under physiological conditions.

Large-strain rheological measurements provide essential information about the structural integrity and extensibility of mucus, providing a more comprehensive understanding of its behaviour in realistic, dynamic scenarios. This understanding is vital, as physiological processes like coughing and cilia beating subject mucus to significant stress and deformation which need to be over the critical stress and deformation for effective clearance. For instance, while the shear stress generated by cilia beating is around 0.7 Pa^1^, which is sufficient to transport healthy mucus, this is far from enough to dislodge mucus in obstructive diseases. In diseases like CF and NCFB, we have shown that the critical shear stress required to initiate mucus clearance is over 10 Pa and can reach 100 Pa in the worst cases. Moreover, during coughing, shear stresses of up to 100 Pa^1,15^ have been calculated for healthy individuals with high cough velocities, but such values are rarely attainable in patients with obstructive lung diseases. For lower cough velocities, the estimated shear stress often falls to around 1 Pa^15^ or less. Importantly, 1 Pa of shear stress is insufficient to clear mucus from the airways of patients with muco-obstructive pulmonary diseases. This underscores the necessity of large-strain rheological analysis, as it better reflects the mechanical forces required to clear mucus in disease states, offering deeper insights into the challenges of therapeutic interventions for effective mucus removal.

## Conclusion

In this study, we demonstrate that macrorheology with cone-plate geometries is a reproducible method for analysing 86 µL of mucus obtained from epithelial ALI cell culture. The investigation of mucus rheological behaviour is crucial to comprehend respiratory functioning and to improve and develop treatments for obstructive diseases. Within this context, ALI cultures have emerged as a valuable model for exploring the rheological characteristics of mucus, aiming to enhance our understanding and fine-tune the interactions within its structure.

The lower γ_c_ and tan δ found for ALI mucus indicate that it is more responsive to external forces and undergoes deformation more easily due to a more liquid conformation, thus less contact between the molecules. Overall, sputum samples demonstrate a more extensible structure compared to the more fluid-like and less structured ALI mucus.

Comprehensive studies that encompass not only concentration-dependent low deformation rheology but also explore the large deformation region, where structural and molecular interactions exhibit clear differences, are essential for gaining a profound understanding of the intricate viscoelastic network. Moreover, an exploration of the swelling dynamics of the mesh can shed light on the reversibility of connections within the network, providing valuable insights into the pathophysiology of obstructive diseases. This knowledge can guide improvements in treatment penetration, offering new perspectives for the development of more effective therapeutic interventions.

In summary, the versatile nature of ALI mucus as a research model offers a valuable platform for unravelling the complexities of mucus rheology and structure. These insights contribute to our broader understanding of respiratory conditions, paving the way for targeted therapeutic interventions and advancements in patient care. Furthermore, the importance of non-linear mucus rheology has been highlighted in our results as it is a clear marker of structural alterations and its study merges as a critical tool for delving deeper into the dynamic and complex nature of mucus.

## Data Availability

The datasets used and/or analysed during the current study are available from the corresponding author on reasonable request.
